# Serotype replacement and mobile genetic elements in Streptococcus pneumoniae: a systematic review

**DOI:** 10.1099/mgen.0.001497

**Published:** 2025-09-09

**Authors:** Gabriel Temitope Sunmonu, Stephanie W. Lo, Anna E. Sheppard, Abiodun David Ogunniyi

**Affiliations:** 1 School of Animal and Veterinary Sciences, The University of Adelaide, Roseworthy, South Australia 5371, Australia; 2 Australian Centre for Antimicrobial Resistance Ecology, The University of Adelaide, Adelaide, South Australia, Australia; 3 Parasites and Microbes, Wellcome Sanger Institute, Hinxton, UK; 4 Milner Centre for Evolution, Department of Life Sciences, University of Bath, Bath, UK; 5 School of Biological Sciences, The University of Adelaide, Adelaide, South Australia 5005, Australia

**Keywords:** antimicrobial resistance, mobile genetic elements, serotype replacement, *Streptococcus pneumoniae*

## Abstract

*Streptococcus pneumoniae* causes otitis media and severe diseases including pneumonia, meningitis and bacteraemia. The rise of antimicrobial resistance (AMR) in *S. pneumoniae*, facilitated by mobile genetic elements (MGEs), complicates infection treatment. While pneumococcal conjugate vaccine (PCV) deployment has reduced disease burden, non-vaccine serotypes (NVTs) have increased and now cause invasive disease. Although PCV reduced the overall AMR incidence, AMR prevalence among NVT pneumococci has increased, creating dual challenges of MGE-driven AMR spread and serotype replacement. In this review, we analysed geographical patterns of serotype replacement and the role of MGE-driven AMR in *S. pneumoniae* using predefined search terms related to pneumococcus, MGEs and serotype replacement. Search outputs were managed through COVIDENCE. We de-duplicated 3,634 articles, screened 2,085 by title/abstract, assessed 423 based on exclusion criteria, reviewed 298 full texts and included 70 studies meeting our inclusion criteria. Global data revealed reductions in vaccine serotypes following vaccination, with concurrent NVT increases. Tn*916*-like and Tn*5253*-like integrative and conjugative elements (ICEs) were associated with tetracycline and macrolide resistance mobilization. Multidrug-resistant NVTs (15A, 15C, 23A, 34 and 35B) continue emerging globally. Our analysis further reinforces other findings that while PCV implementation has successfully reduced vaccine serotype pneumococcal prevalence globally, this success is accompanied by substantial serotype replacement across all continents. This shifting landscape is further complicated by the widespread presence of MGEs mediating AMR in both vaccine and NVTs, particularly through Tn*916*-like and Tn*5253*-like ICEs. These dual challenges underscore the urgent need for improved antimicrobial stewardship programmes and the development of serotype-independent vaccines.

**Impact Statement**

The inexorable rise of antimicrobial resistance (AMR) in *Streptococcus pneumoniae*, driven by horizontal gene transfer via mobile genetic elements (MGEs), has created significant treatment challenges for pneumococcal infections. While conjugate vaccines have substantially reduced disease incidence and overall AMR, the prevalence of AMR among non-vaccine serotypes has increased. The combined challenges of AMR spread through MGEs and serotype replacement complicate efforts to effectively manage *S. pneumoniae* infections. Our systematic review of the global geographical patterns of serotype replacement and MGEs reveals that vaccine introduction has led to a significant reduction in vaccine serotypes, but with a concurrent rise in non-vaccine serotypes. Furthermore, Tn*916*-like and Tn*5253*-like integrative and conjugative elements were linked to the mobilization of tetracycline and macrolide resistance determinants in *S. pneumoniae*. These findings highlight the pressing need for the implementation of comprehensive antimicrobial stewardship programmes to ensure the long-term effective management of pneumococcal infections through the deployment of serotype-independent vaccines to reduce the overall incidence of pneumococcal disease.

## Data Summary

All supporting data are available within the article or in the supplementary files, including the list of publicly available articles reviewed in this paper.

## Introduction

The upper respiratory tract of humans naturally harbours *Streptococcus pneumoniae* (the pneumococcus) and other commensal bacteria as part of the normal flora that can colonize the nose and throat without causing illness [1]. The prevalence of asymptomatic carriage can vary among different individuals and populations [2]. However, while *S. pneumoniae* can exist as a commensal bacterium in the nasopharynx, it can also cause serious illness, especially in individuals with weakened or immature immune systems. Globally, *S. pneumoniae* is the leading cause of acute otitis media and invasive pneumococcal diseases (IPDs), including pneumonia, bacteraemia and meningitis [2,3]. In such instances, the pneumococcus can translocate from the nasopharynx to the lungs and, in some cases, to other regions of the body such as the blood, brain or middle ear. The evasion of host defences due to the host’s weakened immune system explains why young children and the elderly experience a higher rate of pneumococcal infection [1,4]. Moreover, *S. pneumoniae* is a common cause of pneumonia following influenza infection [5].

The capsular polysaccharide of *S. pneumoniae* is a major surface virulence factor that contributes significantly to disease pathogenesis. Over 100 serotypes have been identified to date, distinguished by differences in capsule structure that arise from variations in the genes of the capsule synthesis locus (*cps*) [6]. The 23-valent pneumococcal polysaccharide vaccine (PPV23) consists of purified preparations of the 23 most prevalent capsular serotypes associated with disease in adults. The PPV23 is not conjugated to a protein carrier and therefore only induces a short-term protective antibody response and is not effective in children [7,8]. At the beginning of the 1980s, 85–90% of invasive pneumococcal infections worldwide were caused by the 23 serotypes that the PPV23-based vaccine targeted (Table 1) [9,10]. The PPV23 is given to the elderly and to individuals 2 years or older with underlying medical conditions or increased risk for pneumococcal disease. However, there is still debate regarding its effectiveness against pneumococcal illnesses, such as pneumonia, and how long the protection imparted lasts [11,12].

**Table 1.** Serotypes contained in pneumococcal vaccines

Vaccine	Serotypes
PCV7	4, 6B, 9V, 14, 18C, 19F, 23F
PCV10	1, 4, 5, 6B, 7F, 9V, 14, 18C, 19F, 23F
PCV13	1, 3, 4, 5, 6A, 6B, 7F, 9V, 14, 18C, 19A, 19F, 23F
PCV15	1, 3, 4, 5, 6A, 6B, 7F, 9V, 14, 18C, 19A, 19F, 22F, 23F, 33F
PCV20	1, 3, 4, 5, 6A, 6B, 7F, 8, 9V, 10A, 11A, 12F, 14, 15B, 18C, 19A, 19F, 22F, 23F, 33F
PPV23	1, 2, 3, 4, 5, 6B, 7F, 8, 9N, 9V, 10A, 11A, 12F, 14, 15B, 17F, 18C, 19A, 19F, 20A, 22F, 23F, 33F
Non-PCV/PPV	6C, 6D, 6E, 6F, 6G, 6H, 7A, 7B, 7C, 7D, 9A, 9L, 10B, 10C, 10D, 10F, 11B, 11C, 11D, 11E, 11F, 12A, 12B, 13, 15A, 15C, 15D, 15F, 16A, 16F, 17A, 18A, 18B, 18F, 19B, 19C, 20B, 21, 22A, 23A, 23B, 24A, 24B, 24C, 24F, 25A, 25F, 27, 28A, 28F, 29, 31, 32A, 32F, 33A, 33B, 33C, 33D, 33E, 33G, 34, 35A, 35B, 35C, 35D, 35F, 36A, 36B, 37, 38, 39, 40, 41A, 41F, 42, 43, 44, 45, 46, 47A, 47F, 48

In order to enhance protection against infections caused by multiple serotypes and to provide T-cell help to increase immunological memory, pneumococcal conjugate vaccines (PCVs) were developed by conjugating pneumococcal capsular polysaccharides to a protein carrier [3]. PCVs were initially developed to target the most prevalent serotypes associated with diseases in children. Subsequently, serotypes commonly resulting in adult infections were added to the formulation [13]. As such, the 7-valent pneumococcal conjugate vaccine (PCV7), which protects against seven serotypes, was first developed, followed by PCV10, PCV13, PCV15 and PCV20, which protect against 10, 13, 15 and 20 disease-causing serotypes, respectively (Table 1) [14]. Although newer vaccines have been developed, PCV13 remains the most widely and routinely deployed at the time of writing [15].

The World Health Organization has identified antimicrobial resistance (AMR) as a global health threat, and *S. pneumoniae* is among the top 10 bacterial pathogens for which AMR is a concern [16,17]. A variety of factors have been linked to the rapid evolution, selection and spread of AMR in and among microbes. Mobile genetic elements (MGEs) such as transposons and integrative conjugative elements (ICE) are one of the drivers of AMR gene mobility in bacteria. These MGEs spread AMR genes across different plasmids and host bacteria. Rarely do *S. pneumoniae* genomes carry plasmids [18], and among the first ICEs found in *S. pneumoniae* were Tn*916* [19] and Tn*5253* [20].

**Fig. 1.**
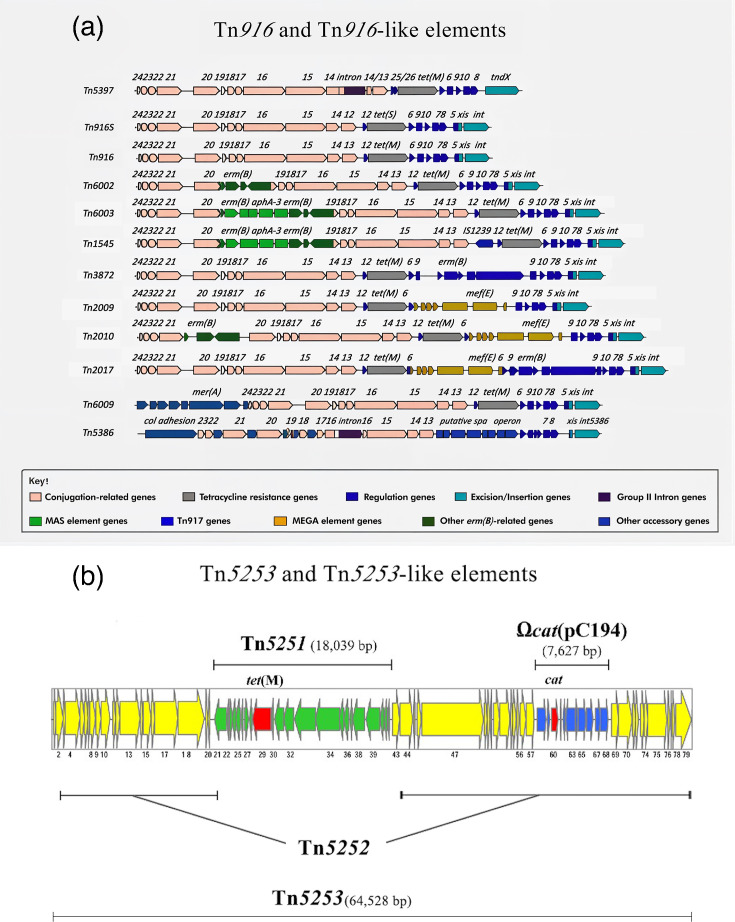
A schematic representation of MGEs associated with AMR in *S. pneumoniae*. (a) Structure of Tn*916* and Tn*916*-like elements (adapted from Roberts and Mullany [104]). The key provides the ORFs’ putative function or origin (MAS represents macrolide-aminoglycoside-streptothricin). (b) Structure of Tn*5253* and Tn*5253*-like elements (adapted from Iannelli *et al*. [21]). Tn*5253* is a 64,528 bp element containing 79 ORFs. Tn*5253* is formed by the integration of the 18,033-bp-long ICE Tn*5251* carrying *tet*(M) into another ICE Tn*5252* carrying the 7,627-bp-long element Ω*cat*(pC194) containing *cat*. AMR genes [*tet*(M) and *cat*] are indicated by red arrows, while other ORFs within Tn*5251*, Ω*cat*(pC194) and Tn*5252* are indicated by green, blue and yellow arrows, respectively.

Tn*916* is an 18 kb conjugative element and was the first ICE reported to encode antibiotic resistance determinants. It carries the tetracycline resistance gene, *tet*(M). Many variants of Tn*916* have been reported (Fig. 1a) [19]. Tn*5253* is a 64.5 kb element that includes the Ω*cat*(pC194) element, which carries the chloramphenicol resistance (*cat*) gene, and Tn*5251*, a Tn*916*-like ICE harbouring *tet*(M) (Fig. 1b) [21]. The sequence beyond Tn*5251* within Tn*5253* is named Tn*5252*. These ICEs (Tn*916*-like and Tn*5253*-like) are characterized by their ability to integrate into the bacterial chromosome and facilitate their own conjugative transfer between bacterial cells. The overall sequence homology of these ICEs is the main reason for their classification as Tn*916*-like and Tn*5253*-like. While they may have variations in accessory genes, the core structure and genes involved in integration, excision and transfer are conserved.

Multidrug-resistant (MDR) *S. pneumoniae* displays global prevalence with varying geographical patterns [22]. MDR in *S. pneumoniae* is usually defined by resistance to at least three classes of antibiotics (usually *β*-lactams, tetracyclines and macrolides). Resistant genetic lineages are the source of most MDR pneumococcal isolates, with a small percentage of these lineages accounting for the majority of MDR pneumococcal isolates globally [22].

In this review, we sought to investigate the role of serotype replacement and MGE-mediated AMR in *S. pneumoniae* as this is crucial for the development of effective strategies required to combat pneumococcal infections.

## Methods

### Search strategy and eligibility criteria

COVIDENCE was used to fulfil the checklist from the Preferred Reporting Items for Systematic Reviews and Meta-Analyses (PRISMA) statement [23]. Before reviewing the literature, the eligibility criteria were established to enable a thorough systematic search. We included all epidemiological, clinical and surveillance studies that reported serotype distribution or prevalence both before and after vaccination, as well as studies on MGEs associated with AMR in pneumococcus. Studies were included regardless of location, age group, disease type or identification method. Brief reports and studies that reported original data were also included, while reviews, animal studies, cloning experiments or studies on MGE without AMR were excluded.

A systematic review of the literature was conducted across three databases (PubMed, Scopus and Web of Science) using the specific search term detailed in Table 2.

**Table 2.** Search strategy (12 December 2023)

Search terms	PubMed	Web of Science	Scopus
((‘Streptococcus pneumoniae’) OR (‘pneumococcus’)) AND ((‘mobile genetic element’) OR (‘plasmid’) OR (‘integron’) OR (‘insertion sequence’) OR (‘transposon’) OR (‘integrative conjugative element’) OR (‘integrative and conjugative element’) OR (‘serotype replacement’))	840	1,210	1,584

### Study selection

Outputs from the literature search from each database were transferred to COVIDENCE. Identified articles were first automatically de-duplicated and then manually de-duplicated. Screening for inclusion was conducted in two phases: (i) title and abstract and (ii) full-text review. Studies on MGE without the context of AMR, studies lacking information on serotype replacement/prevalence before and after PCV vaccination, review articles, animal studies, cloning experiments and mathematical modelling studies were manually excluded.

In the meta-analysis, we included epidemiological, clinical and surveillance studies that reported serotype distribution for pneumococcus before and after vaccine administration in both carriage and IPD from children and adults for studies on serotype replacement/prevalence. Studies were eligible for inclusion if they reported distribution in raw figures or percentages. We included studies conducted in settings where PCV7, PCV10 or PCV13 had been introduced, as well as studies where PCV7 was introduced into the immunization programme and subsequently replaced by PCV10 or PCV13. Additionally, we included studies from settings where PCV10 or PCV13 was introduced without prior use of PCV7.

For studies on MGE-mediated AMR in pneumococcus, we included studies that used either PCR or sequencing methods to identify the MGEs and resistance genes carried by pneumococcus in the analysis.

### Data collection

Based on PCV introduction, we grouped isolates into two vaccine eras:

- *Pre-PCV era*: Period before the introduction of any PCV in the population.
- *Post-PCV era*: Period starting 3 years after the introduction of the most recent PCV administered to the population.

Serotypes were grouped into two categories:

- *Vaccine serotypes (VTs)*: Serotypes included in the most recent PCV administered to the population.
- *Non-vaccine serotypes (NVTs)*: Serotypes not included in the most recent PCV administered to the population.

We also identified serotypes commonly reported for both carriage and IPD in studies from each continent during pre-PCV and post-PCV eras. Data for VT and NVT during pre-PCV and post-PCV eras, vaccine types, MGEs and AMR gene information were extracted from identified publications into an electronic spreadsheet (Microsoft Excel) for analysis.

### Statistical analysis

For studies that reported for more than one country, we used the raw data from the individual countries as a separate entry.

We created a forest plot to display raw prevalence by serotype category and computed the prevalence differences with 95% CI, both overall and by pooled regional prevalence. We used random effects meta-analysis since we assumed that the indirect serotype replacement effect could vary across countries [24].

We assessed statistical heterogeneity by estimating the between-study variance by using 𝜏^2^ [25]. Heterogeneity between countries was considered low if 𝜏^2^<0.2, fairly reasonable for 𝜏^2^ 0.2–0.5 and fairly high for 𝜏^2^>0.5 and <1.0 [26]. A *P*-value<0.05 was considered statistically significant. Statistical analyses were performed using Stata version 18.5 (StataCorp, https://www.stata.com).

## Results

### Study selection

**Fig. 2.**
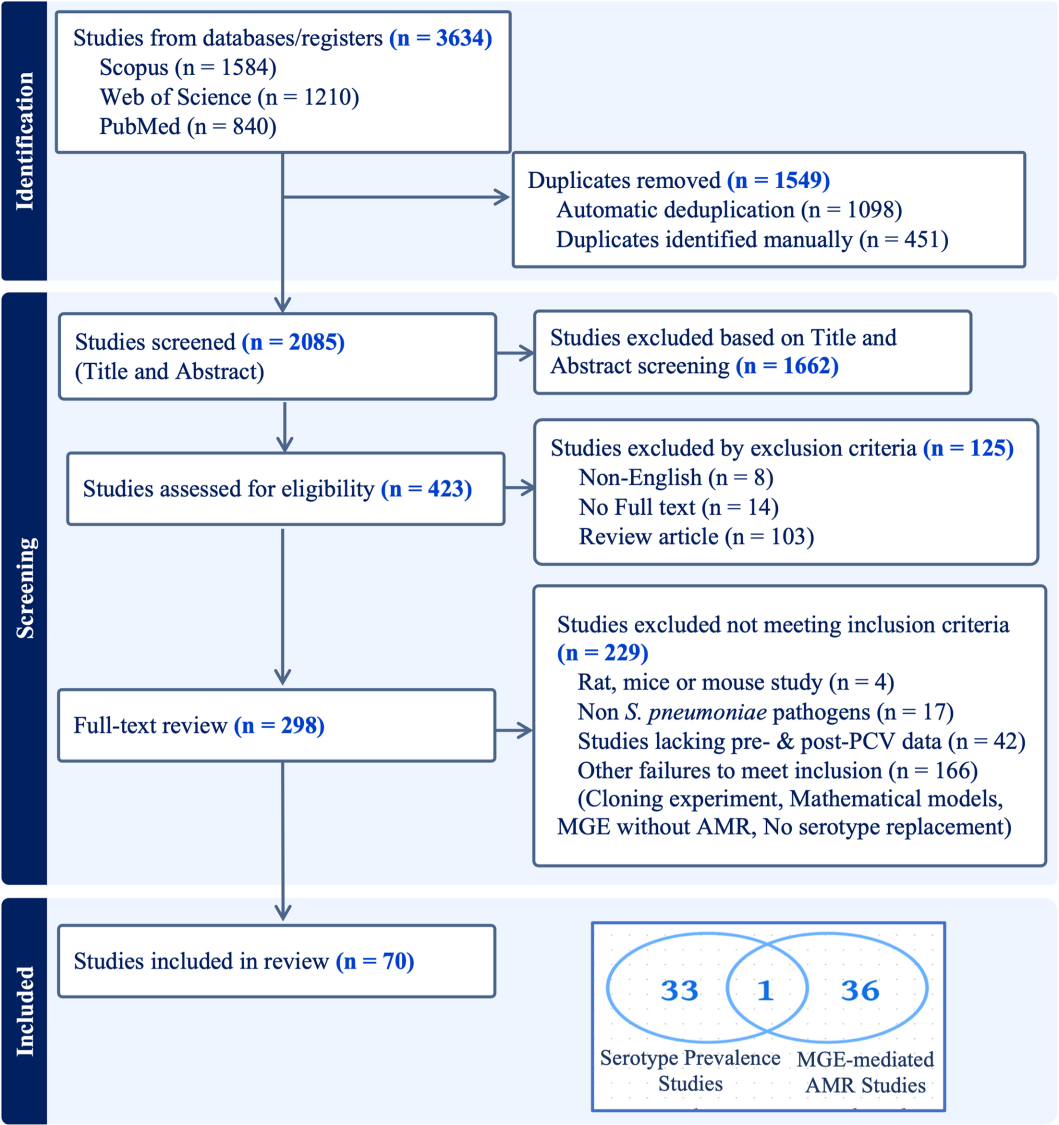
PRISMA flow diagram of literature reviewed.

We identified 3,634 articles, which were first automatically and then manually de-duplicated. After screening titles and abstracts of the remaining 2,085 articles, 423 articles were selected for further assessment based on exclusion criteria. Of these, we were able to retrieve and review 298 full-text publications, ultimately including 70 studies (Fig. 2 and Table S1, available in the online Supplementary Material) meeting our inclusion criteria.

### Study characteristics

The publication years of the included papers range from 2000 to 2022. Of the included papers, 33 reported serotype prevalence during pre-PCV and post-PCV periods, 36 reported MGE-mediated AMR and 1 reported both serotype prevalence and MGE-mediated AMR from Spain (Figs 2 and S1).

**Fig. 3.**
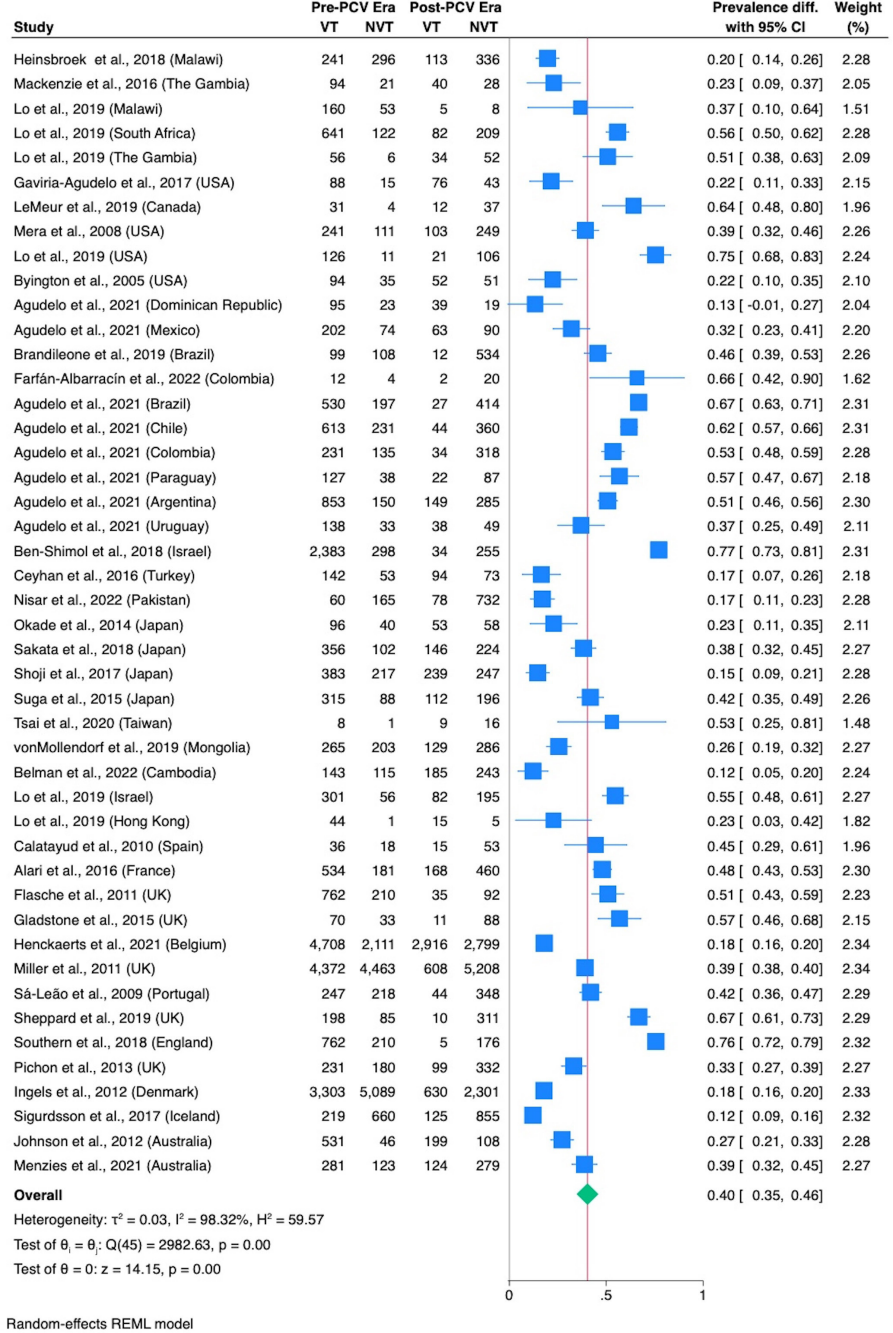
Forest plot and meta-analysis for serotype prevalence pre- and post-PCV era. For each study, the estimated prevalence difference with the corresponding 95% CI and the result of random-effects meta-analysis is shown. 𝜏², the absolute variance in the true effect sizes across studies; I^2^, the estimated between-study heterogeneity.

For the papers describing serotype prevalence, two [27,28] reported data from multiple countries. Lo *et al*. [27] covered six countries (Malawi, South Africa, The Gambia, USA, Israel and Hong Kong) across three regions (Africa, North America and Asia), while Agudelo *et al*. [28] covered eight countries (Dominican Republic, Mexico, Brazil, Chile, Colombia, Paraguay, Argentina and Uruguay) across two regions (North America and South America). The remaining papers were from Africa (*n*=2), North America (*n*=4), South America (*n*=2), Asia (*n*=10), Europe (*n*=12) and Oceania (*n*=2). Each country from a study represented one entry, yielding 46 data points. Countries with multiple entries included the UK (*n*=5), Japan (*n*=4), USA (*n*=4), The Gambia (*n*=2), Malawi (*n*=2), Brazil (*n*=2), Colombia (*n*=2), Israel (*n*=2) and Australia (*n*=2) (Fig. 3 and Table S2). The type of vaccine, collection year, sample type (disease/carriage), age and number of VT and NVT pre- and post-PCV are presented in Table S2.

For the papers describing pneumococcal isolates carrying AMR genes driven by different MGEs, four [29,32] reported MGE-mediated AMR from multiple countries; hence, aggregated data for their entire collections were analysed. The remaining papers were from Africa (*n*=2), North America (*n*=2), South America (*n*=1), Asia (*n*=11), Europe (*n*=16) and Oceania (*n*=1) totalling 36 data points. The most represented countries were Italy (*n*=7), Spain (*n*=6), China (*n*=4), USA (*n*=2), UK (*n*=2), Iran (*n*=2) and Japan (*n*=2) (Table S3). The collection year, study type (random/resistance focused), number of isolates, sample type (disease/carriage), detection methods, AMR genes and MGEs are presented in Table S3.

### Pneumococcal serotype prevalence

#### Pooled regional analysis

**Fig. 4.**
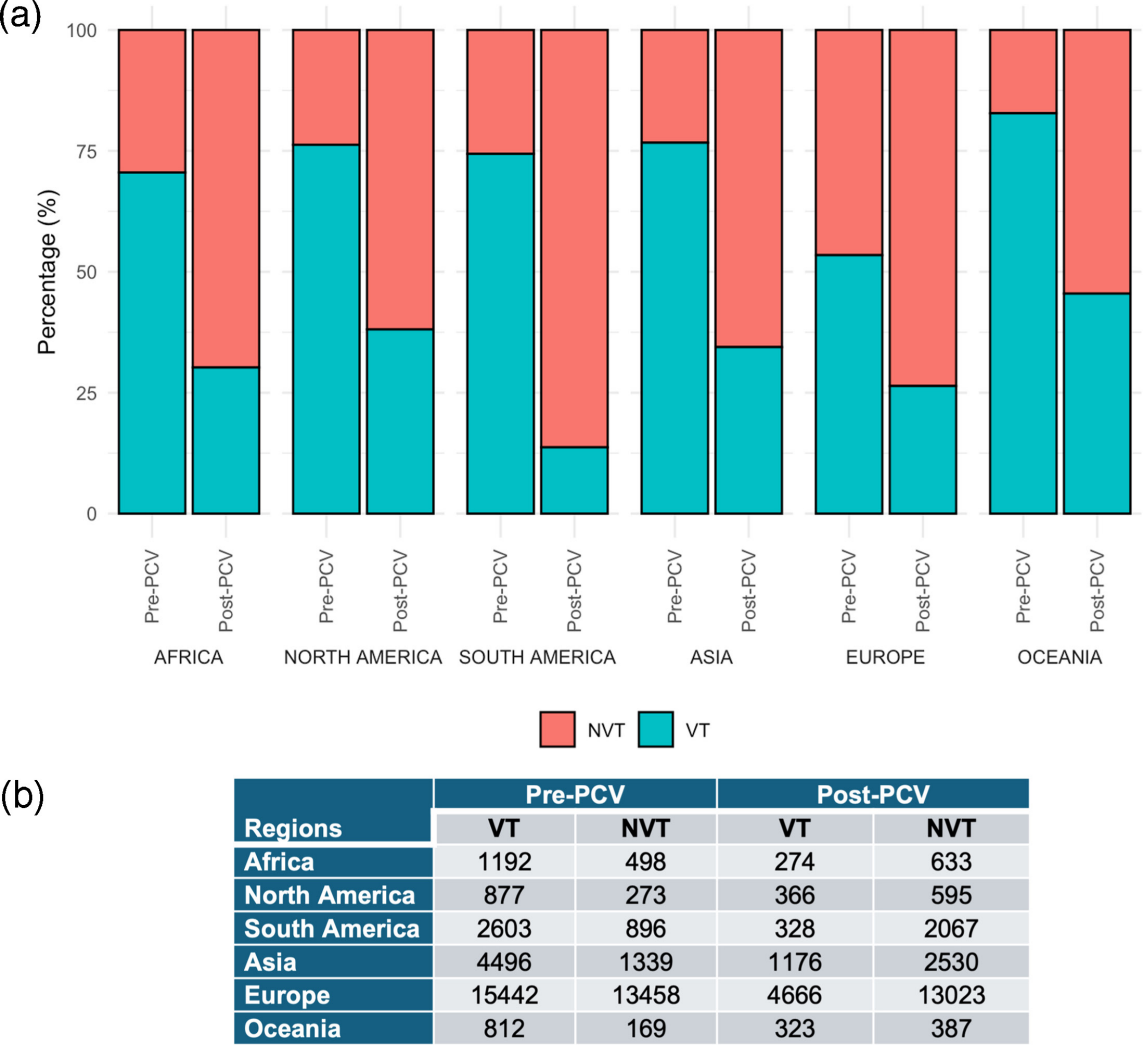
Pooled regional VT and NVT distribution pre- and post-PCV in (**a**) percentages and (**b**) pooled prevalence.

The distribution of pneumococcal serotypes exhibits geographic heterogeneity. This pattern reflects evolutionary processes including geographical isolation, random genetic drift and selective pressures from regional vaccination programmes and their varying coverage. Overall, all regions showed a consistent pattern of VT decline after PCV introduction. This is accompanied by an increase in NVT prevalence (Fig. 4), suggesting serotype replacement. The most prevalent serotypes in different regions before and after PCV administration are presented in Table 3 (in detail in Table S2). Even after PCV administration (in most cases, PCV13), several serotypes included in the vaccine remain prevalent globally. Serotype 19A is still prevalent in all regions, while serotype 3 is present in North America, South America, Asia and Europe. Similarly, serotype 6A remains common in South America, Asia and Oceania.

**Table 3.** Most prevalent pneumococcal serotypes

Continent	Era	Serotypes		
		PCV7	PCV13 non 7	Non-PCV13
Africa	Pre-PCV	14, 23F	1, 5, 6A, 19A	–
	Post-PCV	19F, 23F	1, 6A, 19A	15B/C, 16F, 35B
North America	Pre-PCV	6B, 14, 19F, 23F	6A, 19A	–
	Post-PCV	–	3, 6A, 19A	6C, 15B/C, 23A, 35B
South America	Pre-PCV	6B, 14, 23F	1, 3, 6A, 19A	–
	Post-PCV	–	3, 6A	6C, 15A, 15B/C
Asia	Pre-PCV	6B, 14, 19F, 23F,	3, 6A	–
	Post-PCV	19F	3, 6A, 19A	15A, 15B/C, 35B
Europe	Pre-PCV	6B, 14, 19F, 23F	6A, 19A	–
	Post-PCV	19F	3, 19A	8, 15A, 15B/C
Oceania	Pre-PCV	4, 6B, 14, 19F, 23F	5	–
	Post-PCV	–	1, 7F, 19A	6C, 15B/C, 18, 35B

#### Heterogeneity testing

In the global, regional and pooled regional analyses, 𝜏^2^ was <0.2 in all analyses (Figs 3 and S2–S8), suggesting that heterogeneity is low [25].

#### African studies

In Africa, post-PCV, the percentage of VT decreased from 70.5% to 30.2% while NVT increased from 29.5% to 69.8% of total cases (Fig. 4). African countries primarily implemented PCV13, with countries including The Gambia [27,33] and South Africa [27] using a sequential PCV7 to PCV13 implementation, while Malawi only implemented PCV13 [27,34]. The most prevalent serotypes pre-PCV were serotypes 1, 5, 6A, 6B, 12F, 14, 15B/15C, 19A and 23F, while post-PCV, serotypes 1, 12F, 15B/15C and 35B/35D were most prevalent [27,33, 34]. Some of the most common NVTs in Africa post-PCV include 13, 15B/C, 16F and 35B in IPD cases, mostly in children [33,35], and 13, 15B/C, 16F, 23A/B and 35B in pneumococcal carriage in children [36].

#### North American studies

In North America, post-PCV, the percentage of VT decreased from 76.3% to 38.1%, while NVT increased from 23.7% to 61.9% of total cases (Fig. 4). Two US studies [37,38] reported implementation of PCV7 only, while two more recent studies [27,39] described a sequential transition from PCV7 to PCV13. In Canada, one study [40] reported PCV13 implementation following initial PCV7 and PCV10 administration. In the Dominican Republic and Mexico, PCV13 was administered without any prior PCV usage [28]. In the USA, PCV7 serotypes decrease after the introduction of the PCV7 vaccine, with a simultaneous increase in non-PCV7 serotypes [39]. A similar trend was observed following PCV10 introduction in Canada [40]. The subsequent introduction of PCV13 led to a reduction in PCV13 serotypes. However, non-PCV13 serotypes (such as serotypes 6C, 9N/L, 12A, 23A and 23B) became more prevalent [39,41]. The study [40] from Canada reported on the introduction of PCV7 and the early mass adoption of PPV23, which initially reduced outbreaks of serotype 1, but IPD due to serotypes covered by PPV23 continues to occur, highlighting its short-lived protection. Subsequent introduction of PCV10 and PCV13 reportedly led to a decrease in serotypes covered by these vaccines. The most prevalent serotypes pre-PCV were serotypes 3, 6A, 6B, 12F, 14, 19A, 19F and 23F, while post-PCV, serotypes 3, 15B, 19A, 23B and 35B/35D were most prevalent. Overall, some of the most common NVTs in the Americas after PCV administration causing IPD include 6C, 7C, 11A, 23A and 23B, mostly in children [39,42, 43], and 33F and 16F in adults [42]. Similarly, NVT reported in pneumococcal carriage in children, including 6C, 11A, 15B, 16F, 23A and 23B, was reported [44].

#### South American studies

In South America, post-PCV, the percentage of VT decreased from 74.6% to 38.1%, while NVT increased from 25.4% to 86.3% of total cases (Fig. 4). In South America, PCV10 was administered in Brazil [28,44], Colombia [28,44], Chile [28] and Paraguay [28], while PCV13 was implemented by Argentina [28] and Uruguay [28]. The most prevalent serotypes pre-PCV were serotypes 1, 3, 6A, 6B, 14, 19A and 23F, while post-PCV, serotypes 3, 6A, 6C, 15A, 15B/15C and 24F were most prevalent [28,44].

#### Asian studies

In Asia, post-PCV, the percentage of VT decreased from 77.1% to 31.7%, while NVT increased from 22.9% to 68.3% of total cases (Fig. 4). PCV7 followed by PCV13 was administered in Israel [27,45], Turkey [46], Taiwan [47] and Hong Kong [27]. PCV10 was administered in Pakistan [48], while PCV13 was used in Mongolia [49] and Cambodia [50]. Three studies [51,53] reported prevalence after PCV7 administration in Japan, while one [54] reported after PCV13 administration in Japan. Studies that reported the initial introduction of PCV7 in Asia reported a peak in the prevalence of PCV7 serotypes just before the introduction of the PCV7 vaccine, which were then replaced by PCV13 serotypes. The PCV13 serotypes became dominant until the introduction of the PCV13 vaccine, after which there was a decline in prevalence of PCV13 serotypes except for serotype 3 [54]. Despite the reduction in VT, NVT, such as 6C, 15A, 22F, 23A, 34, 35A and 35B, became more prevalent and replaced the VT [27,52, 53]. Overall, in Asia, several NVTs are commonly reported. For IPD cases, especially in children, serotypes 6C, 15A, 23A and 24F are prevalent [27,52, 53]. Among adults with IPD, serotypes 6C, 15A, 23A and 35B are frequently reported [54]. In pneumococcal carriage studies involving children, serotypes 11A, 15A, 15F, 23A and 23B are commonly reported [47,48].

#### European studies

In Europe, post-PCV, the percentage of VT decreased from 53.4% to 26.4%, while NVT increased from 46.6% to 73.6% of total cases (Fig. 4). Six studies reported the administration of PCV7 only in Spain [55], UK (*n*=3) [56,58], Portugal [59] and Denmark [60]. The transition from PCV7 to PCV13 was reported in France [61] and UK (*n*=3) [62,64]. PCV10 and PCV13 were introduced in Iceland [65] and Belgium [66], respectively. Studies that investigated serotype prevalence after the introduction of PCV7 reported a decrease in the abundance of PCV7 serotypes and an increase in non-PCV7 serotypes [58,60]. Similarly, a reduction in PCV13 serotypes was reported after the introduction of PCV13, but some PCV13 serotypes (3 and 19A) and the non-PCV13 serotypes 24F, 23B, 10A, 15A and 6C were still prevalent [61]. Generally, in Europe, the incidence of PCV7, PCV10 and PCV13 serotypes (except serotype 3) has declined drastically, while NVTs are on the rise both in IPD and nasopharyngeal carriage among all age groups. Despite the use of PCV13, which is expected to protect against serotype 3, serotype 3 remains highly prevalent, causing a substantial proportion of IPD, especially among adults [14,56, 61]. One study [67] from France investigated serotype distribution before the introduction of vaccines. Both vaccine (3, 6A, 6B, 9V, 14, 18C, 19A, 19F and 23F) and non-vaccine serotypes (11A, 15A, 15B, 23A, 17F, 10A and 9L) were present in relatively equal proportion. Another study [68] from France showed that after the introduction of PCV7, the non-PCV7 serotypes 19A, 7F, 3, 12F, 23B, 24F and 35B became more prevalent. Overall, some of the most common NVTs in Europe after PCV administration causing IPD include 6C, 15A and 24F, mostly in children and 8, 9N and 15A in adults [14,69]. For pneumococcal carriage in children, NVTs 6C, 21, 23B and 35F are commonly reported [64,70, 71].

#### Oceanian studies

In Oceania, post-PCV, the percentage of VT decreased from 82.8% to 45.5%, while NVT increased from 17.2% to 54.5% of total cases (Fig. 4). The two studies [72,73] from Oceania were from Australia and reported on serotypes associated with IPD. In Australia, PPV23 is specifically given to adults. The two studies examined PPV23’s contribution to IPD prevalence: one in conjunction with PCV7 alone [72], and another following both PCV7 and PCV13 sequential introductions [73].

A reduction in VTs causing IPD was reported by the two studies, which was counteracted by replacement with NVTs causing IPD. Despite the introduction of PCV7, PCV13 and PPV23 vaccines, recent reports showed that NVTs (6C, 10F, 13, 15A, 15C, 16F, 18A, 18B, 18F, 22A, 23A, 23B, 33B, 35B, 35F and non-typeable) account for most IPD cases, mostly in children and the elderly [72]. The incidence of IPD due to NVTs was more prominent among indigenous Australians than non-indigenous Australians [72,73].

### MGEs driving AMR in *S. pneumoniae*

Thirty-seven studies reported on the prevalence of MGEs carrying AMR genes in *S. pneumoniae* (Table S3). The MGEs driving AMR in *S. pneumoniae* primarily consist of two ICE groups: Tn*916*-like (Fig. 1a) and Tn*5253*-like (Fig. 1b). The identified Tn*916*-like and Tn*5253*-like ICEs carry genes conferring resistance to one or more of the following antibiotics: tetracycline, macrolide, chloramphenicol and aminoglycoside (Table 4). Some elements in the Tn*916*-like group are composite structures that include smaller, transposable genetic elements that are mobile.

**Table 4.** Characteristics of MGEs responsible for AMR in *S. pneumoniae*

Element	No. of studies	Resistance gene(s)				Reference(s)
		Tetracyclines	Macrolides	Aminoglycosides	Chloramphenicol	
**Tn** *916* **-like group**						
Tn*1116*	2	*tet*M	*erm*B	–	–	[79,85]
Tn*5397*	1	*tet*M	*erm*B	–	–	[55]
Tn*916*	12	*tet*M	–	–	–	[29,31, 32, 75, 76, 81, 82, 84, 86, 87, 105,107]
Tn*6002*	16	*tet*M	*erm*B	–	–	[55,75, 77, 79,81, 84]
Tn*6003* or Tn*1545*	17	*tet*M	*erm*B	*aphA*-3	–	[30,55, 75, 79, 85, 86, 89, 90, 109,117]
Tn*3872*	15	*tet*M	*erm*B	–	–	[55,75, 79,81, 84]
Tn*2009*	10	*tet*M	*mef*E	–	–	[55,75, 79, 81, 85, 86, 90, 107, 118, 119]
Tn*2010*	11	*tet*M	*mef*E, *erm*B	–	–	[55,77, 80, 85, 86, 90, 106,109, 118]
Tn*2017*	3	*tet*M	*mef*E, *erm*B	–	–	[81,86, 106]
Tn*917*	7	–	*erm*B	–	–	[32,79,81, 84, 90, 114]
**Tn** *5253* **-like group**						
Tn*5253*	4	*tet*M	–	–	*cat*	[80,105, 115, 120]
Tn*5252*	3	–	–	–	*cat*	[78,106, 113]
Tn*1311* or SpnRi3*erm*(B)	2	–	*erm*B	–	*cat*	[79,80]
ICE*Sp*23FST81	2	*tet*M	–	–	*cat*	[105,106]
**Others**						
Tn*1207.1*	4	–	*mef*A*, msr*D		–	[55,74, 79, 116]
mega	12	–	*mef*A *mef*E/*mef*I*/msr*D		–	[30,32, 55, 74,76, 78, 79, 81, 90, 107, 116, 118]

Pooled global analysis of MGE driving AMR in *S. pneumoniae*.

mega, Macrolide Efflux Genetic Assembly.

Data from a total of 4,523 resistant *S. pneumoniae* isolates from 37 studies covering over 15 countries were analysed. The most represented countries include Italy (*n*=7), Spain (*n*=6), China (*n*=4), USA (*n*=2), UK (*n*=2), Iran (*n*=2) and Japan (*n*=2). Four studies [29,32] reported on isolates collected from more than one country. Of the total isolates, 78.9% were disease-associated, 12.7% were carriage-associated and 8.4% were reported as a mixture of both, without distinguishing the number of isolates from disease or carriage sources.

The Tn*916*-like group was the most prevalent: Tn*916* (*n*=733), Tn*6002* (*n*=514), Tn*1545*/*6003* (*n*=413), Tn*3872* (*n*=169), Tn*2010* (*n*=138), Tn*917* (*n*=95), *Tn2009* (*n*=54) and *Tn5897* (*n*=28). For the Tn*5253*-like group, 105 isolates carried Tn*5252*, and 33 carried Tn*5253*. Additional MGEs include Macrolide Efflux Genetic Assembly (mega) (*n*=378), Tn*1207.1* (*n*=72), ICE*Sp*23FST81 (*n*=38), Tn*1116* (*n*=19) and Tn*1311*/SpnRi3*erm*(B) (*n*=2). Individual isolates could carry more than one MGE, typically combining either a Tn*916*-like element with mega, a Tn*5253*-like element with mega or a Tn*5253*-like element with a Tn*916*-like element.

Multiple resistance genes frequently co-occur within Tn*916*-like and Tn*5253*-like ICEs. Tn*3872* is predominant in Europe (57.8%) and Asia (28.6%), while Tn*6002* shows the highest prevalence in Europe (63%) and Asia (36%). The *tet*(M)-Tn*916* association appears to be globally conserved, suggesting its fundamental role in tetracycline resistance dissemination, with *tet*(M) showing the highest prevalence within Europe (67%) and Asia (57.5%). Similarly, *erm*(B) is widespread across all continents with prevalence exceeding 70% in most regions. European isolates demonstrate the highest diversity of both AMR genes and MGEs, with *erm*(B)-mediated macrolide resistance predominating over *mef*(A)-mediated resistance, while Asian isolates show distinct patterns characterized by *mef* genes and mega elements. Notably, *mef* genes show higher prevalence in Asia (37.4%) compared to other regions.

#### Macrolide resistance

The macrolide resistance gene *erm*(B) was the most prevalent, found in 41.1% (*n*=1858) of isolates, from 34 studies. Other reported macrolide resistance genes include *mef*(A) (*n*=329) from 15 studies, *mef*(E) (*n*=322) from 16 studies, *mef*(I) (*n*=2) [74] from 1 study and *msr*(D) (*n*=34) from 4 studies [75,78]. The integration of the mega element, which contains *mef*(E), into Tn*916* ICE formed the composite element Tn*2009* linking *mef*(E) and *tet*(M) in *S. pneumoniae* as reported by 11 studies. The Tn*916*-like group of ICE carries almost all (99%, *n*=1858) of *erm*(B), while the remaining two were carried on Tn*1311* or SpnRi3*erm*(B) [79,80]. Similarly, all *mef*(A) and *mef*(E) are carried on mega alone or mega within a Tn*916*-like ICE. The detected *msr*(D) gene was mostly carried by mega (97.1%; 33/34) [75,76, 78] and a Tn*916*-like element (2.9%) [77], while *mef*(I) was not reported to be located on any MGE [74].

#### Tetracycline resistance

Tetracycline (tetracycline, doxycycline) resistance in pneumococcus is mainly caused by ribosomal protection proteins, which attach to the ribosome and displace the antibiotic out of its binding site. The tetracycline resistance gene, *tet*(M), was present in 38.4% (*n*=1739) of all isolates, from 32 studies. Other reported tetracycline resistance genes include *tet*(S/M) (*n*=131) from one study [32], *tet* [31] (*n*=9) from two studies [31,77] and *tet*(K) (*n*=1) [81] and *tet*(L) (*n*=1) [81] from one study. The tetracycline resistance gene *tet*(M) was mostly carried on the Tn*916*-like group (85.1%) and 14.9% on a Tn*916* integrated within a Tn*5253*-like element. In addition, *tet* [31,50, 77] was carried on a genomic island, while *tet*(K) and *tet*(L) were presumed to be carried on mega and Tn*917*+mega, respectively [81]. The *tet*(S/M) carried by pneumococcus was present on a conserved conjugative Tn*916* within ICE*Sp*14ST230, ICE*Sp*14ST156 and ICE*Sp*14ST5359 [32]. ICE-mediated tetracycline resistance was widely disseminated across all continents with corresponding phenotypic evidence.

#### Chloramphenicol resistance

The chloramphenicol resistance gene *cat* was present in 6.4% (*n*=291) of all isolates, from eight studies, with 53.6% (*n*=156) of these genes carried on a Tn*5253*-like ICE (90, 28 and 38 of the genes were carried on Tn*5252*, Tn*5253* and ICE*Sp*23FST81, respectively). Interestingly, 46.4% (*n*=135) of the isolates carrying *cat* were reported to harbour a Tn*916*-like element (possibly from a Tn*5253*-like group carrying *Tn916*) [31,82]. In addition, Tn*3872*, Tn*2009* and no MGE were detected in one isolate each carrying *cat* [80].

#### Aminoglycoside resistance

The aminoglycoside (kanamycin) resistance gene, *aphA*-3, was present in only 42 isolates from 9 studies, with 92.9% (*n*=39) of these genes carried on Tn*1545*/*6003*. Tn*1545* only differs from Tn*6003* by having the insertion sequence IS*1239* between its orf13 and orf12 (Fig. 1a). Two were present within Tn*1311* or SpnRi3*erm*(B) [79,80]. No MGE was reported for one of the isolates carrying the *aphA*-3 gene [75].

#### Non-MGE-mediated resistance

MGEs play a crucial role in disseminating acquired resistance genes among bacteria, facilitating horizontal gene transfer (HGT) between organisms. In *S. pneumoniae*, *β*-lactam antibiotics face resistance primarily through acquisition of low-affinity penicillin-binding protein (PBP), modification of endogenous PBPs through homologous recombination, point mutations or a combination of these mechanisms [83]. Mutations in *pbp2b*, *pbp2x* and *pbp1a* genes confer resistance to *β*-lactam antibiotics in *S. pneumoniae* [22,83]. *β*-lactam resistance-conferring mutations (*pbp2b*, *pbp2x* and *pbp1a*) have been found in *S. pneumoniae* isolates carrying Tn*916*-like ICEs [84], and phenotypic fluoroquinolone resistance has been reported in *S. pneumoniae* isolates carrying Tn*1545* and Tn*916* [30,82]. Resistance mutations in housekeeping genes, including *pbp* (associated with beta-lactam resistance), *folA/P* (linked to cotrimoxazole resistance) and *pmrA*, *parC* and *gyrA* (linked to fluoroquinolone resistance), develop independently of MGEs, as previous studies have not found a significant relationship/correlation between these chromosomal mutations and MGE presence.

### Comparative analysis of MGE burden in VTs vs. NVTs

Analysis revealed that over 80% of isolates carrying MGEs were VT, indicating a disproportionate association between MGEs and vaccine-targeted serotypes. This suggests that MGEs, particularly those conferring AMR, were historically more prevalent in VT populations. Given this VT-MGE association, two post-PCV trends can be speculated: initially, MGE prevalence would be expected to decline following PCV introduction, since VT prevalence falls; however, MGE prevalence could subsequently rise again due to HGT into NVT strains and continued AMR selection pressure.

Pre- and post-PCV stratification showed consistent proportions of MGE-positive VT isolates (81% vs. 82%) (Table S3), suggesting no evidence yet for widespread MGE acquisition by NVT serotypes post-PCV. This finding should be interpreted with caution, as only three post-PCV studies provided post-PCV data disaggregated by serotype, and others lacked sufficient detail to determine MGE distribution across serotypes. The limited post-PCV dataset underscores the need for ongoing genomic surveillance to monitor potential shifts in MGE burden, particularly in NVT populations, where AMR selection could eventually drive new patterns of MGE dissemination.

### Serotypes associated with MDR

Reports of MDR serotypes have emerged globally, but predominantly in Asia and Europe. The most prevalent MDR VT includes 6B, 14, 19F, 19A, 23A and 23F [32,75, 80, 85,89], while the most prevalent NVT includes 6C, 23A and 35F [32,75, 88, 90].

## Discussion and conclusion

The interplay between serotype replacement and the dissemination of MGEs harbouring AMR genes in *S. pneumoniae* presents a difficult challenge in combatting pneumococcal infections. In this systematic review, we present data published from 2000 to 2022 that described serotype replacement and MGEs associated with AMR in *S. pneumoniae*, both in carriage studies and pneumococcal infections.

Widespread deployment of PCVs has correlated with a general reduction in pneumococcal disease caused by VTs. However, after the introduction of PCVs, NVTs increased in prevalence and caused disease. Consequently, the emergence of NVTs exhibiting multidrug resistance remains a significant concern.

All regions demonstrated significant reductions in VT prevalence following PCV implementation. NVT prevalence increased across all regions, suggesting consistent serotype replacement patterns. Initial VTs ranged from 53.4% (Europe) to 82.8% (Oceania). Post-PCV, VTs were 26.4% in Europe and 45.5% in Oceania. This variation in serotype replacement across the different regions might have been influenced by various factors, such as differences in vaccine coverage, local epidemiology and population dynamics, which may have created conditions more favourable for the expansion of NVT after the introduction of PCVs [27,91]. Differences in the extent of serotype replacement might be attributed to regional variations in the prevalence of NVTs before the introduction of PCVs, vaccine schedule, PCV uptake rate and PCV introduction history [92]. Additionally, despite the global reduction in the prevalence of VTs after PCV administration, serotype 3 [93,95] and 19A [91,96, 97] are still circulating widely despite being covered by PCV13.

In Australia, recent data from the Department of Health and Aged Care’s National Notifiable Disease Surveillance System showed that the incidence of IPD has reached its highest peak since 2004. Despite the approval of more effective vaccines for use in the country, many high-risk individuals, especially Indigenous Australians, still face barriers to accessing them. The most recent data indicate that cases of pneumococcal infections rose to 2,268 in 2023 and 2,378 in 2024. These figures represent the highest rates since 2004, leading to demands for the accelerated distribution of a new generation of approved vaccines from the federal government [98,99].

HGT is crucial for bacterial evolution and adaptation. This process enables bacteria to acquire new traits, including those related to disease-causing abilities and resistance to antibiotics. The variety of MGEs that bacteria possess allows them to tap into a vast reservoir of genes. This expanded gene pool significantly enhances bacteria’s capacity to adapt and survive in the face of antimicrobial treatments [100]. MGEs driving AMR in *S. pneumoniae* belong to the Tn*916*-like or Tn*5253*-like ICE groups. Our findings suggest that *tet*(M) has a strong correlation with Tn*916*-like ICE, indicating that this MGE is likely responsible for the mobility of tetracycline resistance. Among these Tn*916*-like ICEs, *erm*(B) is frequently associated with Tn*3872* and Tn*6002*. Similarly, the mega element appears to be linked with *mef* genes. The *tet*(M) gene is usually harboured by these elements except for Tn*5252*, Tn*917*, Tn*1207.1* and Tn*1806*. The elements lacking *tet*(M) carry at least one of *erm*B, *mef*A, *msr*D or *cat*(pC194) [101]. Despite the diverse ICEs found in *S. pneumoniae*, a previous report by Varaldo *et al*. [101] showed that *S. pneumoniae* rarely uses conjugation but has evolved sophisticated transformation systems. Similarly, another study from Nielsen *et al*. [102] showed the co-transfer of *mef*(I) and *catQ* by transformation and not conjugation. Nielsen *et al*. further reported no conjugation in *mef*(E) isolates. Low-frequency conjugation-mediated transfer of *mef*(E) has been reported with both streptococci and staphylococci [103]. These transformation mechanisms enable *S. pneumoniae* to undergo extensive genetic recombination, resulting in remarkable genetic flexibility. This ability to readily incorporate and recombine external DNA is a defining characteristic of *S. pneumoniae*, setting it apart from many other bacterial species and contributing to its adaptability and evolution.

To effectively monitor and respond to these evolutionary changes (AMR and serotype replacement), robust genomic surveillance is critical, including tracking serotype distribution, resistance genes and MGE content in both carriage and disease isolates over time, especially in underrepresented regions. Many studies focus on specific serotypes or lack post-PCV data, limiting generalizability. Expanded, geographically diverse studies are essential.

Our findings underscore the global presence of Tn*916*-like and Tn*5253*-like ICE in *S. pneumoniae*, which carry crucial tetracycline and macrolide resistance determinants. These ICEs harbouring AMR genes are globally disseminated, posing a significant threat to the effectiveness of antimicrobial therapies. This dissemination coincides with a concerning trend: a decrease in the prevalence of vaccine-targeted serotypes, accompanied by the emergence of NVTs on a global scale. This shift (serotype replacement) undermines the efficacy of current pneumococcal vaccines, highlighting the need for serotype-independent vaccines that will not only protect against most serotypes but also reduce the incidence of AMR in pneumococcus.

Despite the wide administration of PCVs across different regions, serotypes 3 and 19A remain prevalent in IPD cases. Both 19A and 19F serotypes carrying MGE-mediated AMR genes are frequently reported. A significant public health concern is that over time, the prevalence of NVT in both carriage and IPD cases has risen following PCV administration. In parallel, MGE-mediated AMR is becoming widespread in pneumococcus, with MDR NVT now emerging. Multifaceted interventions focusing on antimicrobial stewardship, serotype-independent vaccine development, vaccination and infection control are necessary to address the dual challenges posed by serotype replacement and MGE-mediated AMR in pneumococcal infections. By adopting a comprehensive, multifaceted approach that addresses both serotype replacement and MGE-driven AMR, we can make significant strides in combating pneumococcal infections and safeguarding public health. This strategy allows us to strengthen our defences against this formidable pathogen, ensuring a more effective response to the evolving challenges posed by *S. pneumoniae*.

## Supplementary material

10.1099/mgen.0.001497 Uncited Supplementary Material 1.

10.1099/mgen.0.001497 Uncited Supplementary Material 2.

## Abbreviations

AMR: antimicrobial resistance
HGT: horizontal gene transfer
ICEs: integrative and conjugative elements
IPDs: invasive pneumococcal diseases
MDR: multidrug-resistant
mega: Macrolide Efflux Genetic Assembly
MGEs: mobile genetic elements
NVTs: non-vaccine serotypes
PBP: penicillin binding protein
PCV: pneumococcal conjugate vaccine
PCV7: 7-valent pneumococcal conjugate vaccine
PPV23: 23-valent pneumococcal polysaccharide vaccine
PRISMA: Preferred Reporting Items for Systematic Reviews and Meta-Analyses
VTs: vaccine serotypes
