# Supplementary material for: Serotype replacement and mobile genetic elements in Streptococcus pneumoniae: a systematic review
Source: Microb Genom. 2025 Sep 9;11(9):001497. doi: 10.1099/mgen.0.001497 (PMC12452179; doi:10.1099/mgen.0.001497)
Supplement: Uncited Supplementary Material 1. [file mgen-11-01497-s001.pdf]

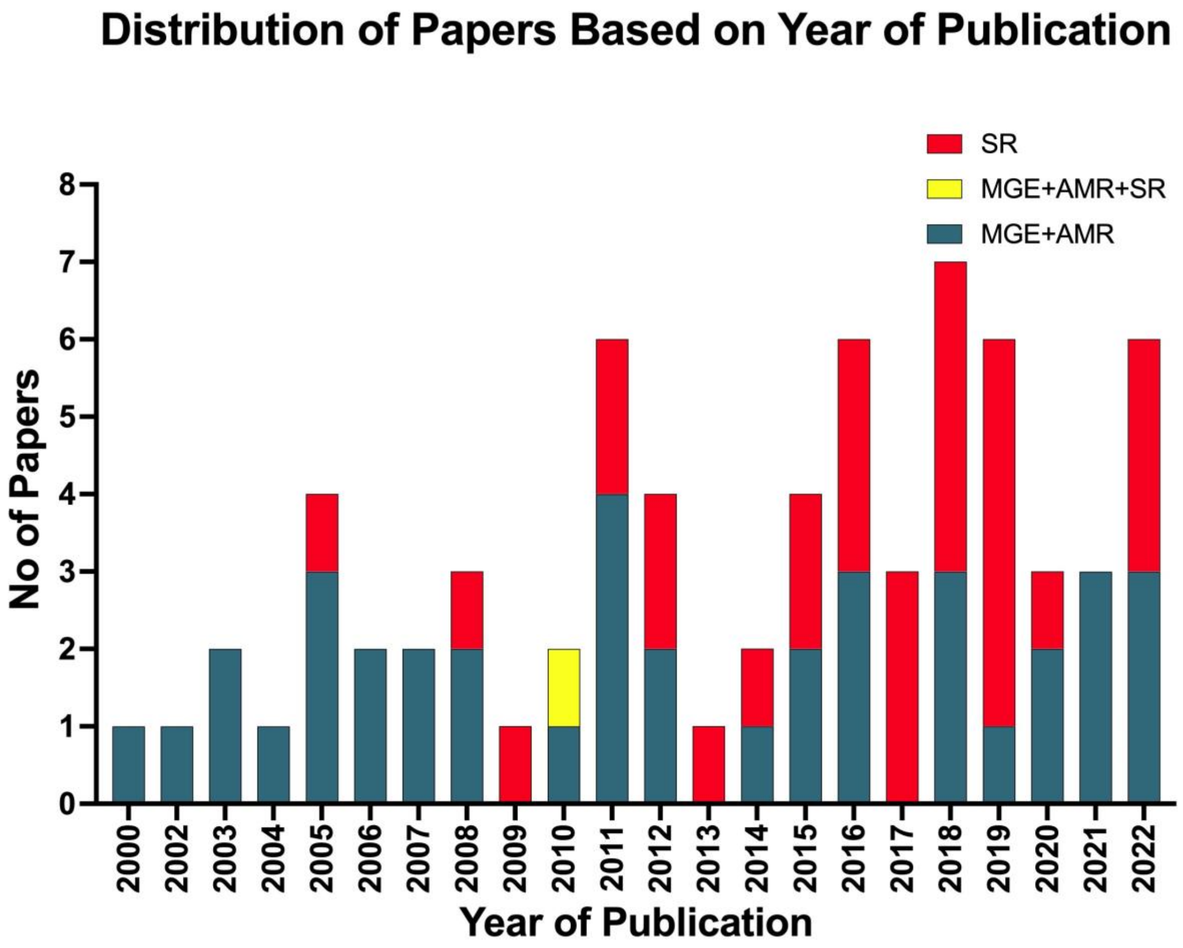

Fig. S1. Number of records included in the study based on year of publication. MGE: mobile genetic element; AMR: antimicrobial resistance; SR: serotype replacement. The yellow bar indicates a publication that reported on both serotype replacement and MGE in the context of AMR by Calatayud et al. 2010.

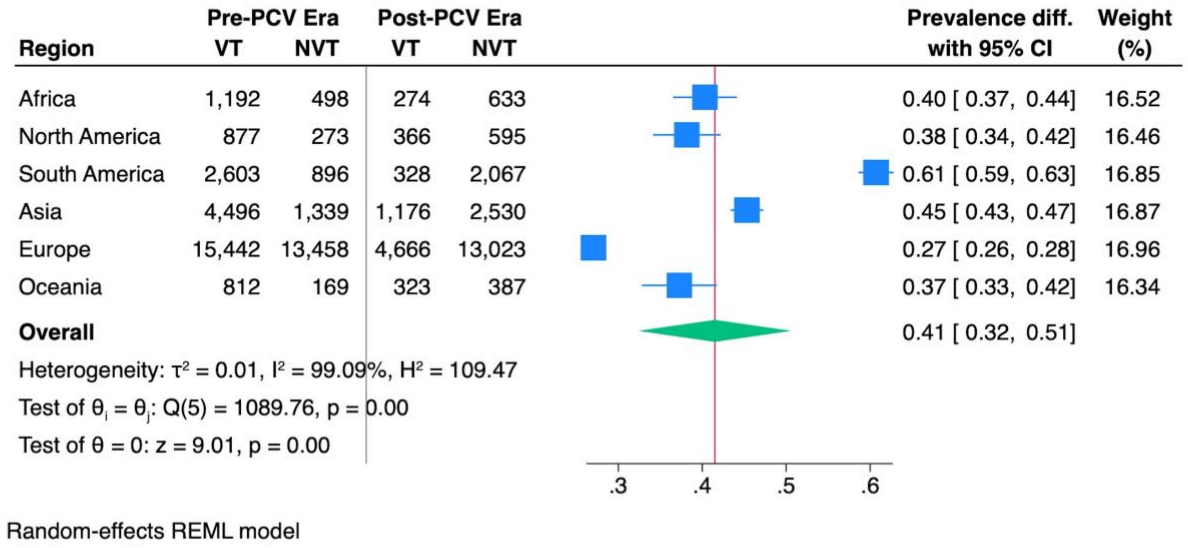

Fig. S2: Pooled forest plot and meta-analysis for serotype prevalence pre and post PCV era in each region. For each study, the estimated prevalence difference with the corresponding 95% confidence interval and the result of random-effects meta-analysis, are shown. VT, vaccine serotypes; NVT, non-vaccine serotypes; τ^2, the absolute variance in the true effect sizes across studies; I2, the estimated between-study heterogeneity.

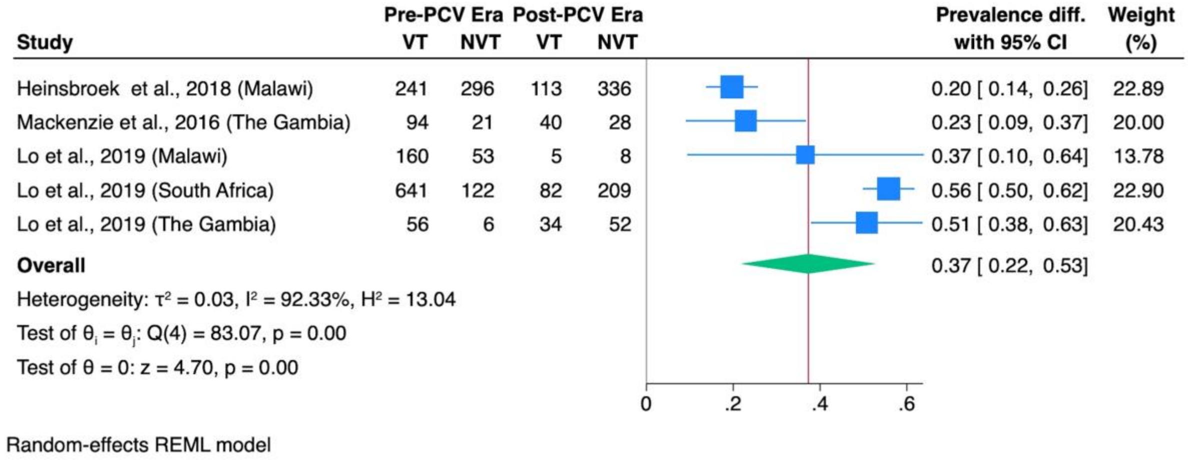

Fig. S3: Forest plot and meta-analysis for serotype prevalence pre and post PCV era in Africa. For each study, the estimated prevalence difference with the corresponding 95% confidence interval and the result of random-effects meta-analysis, are shown. VT, vaccine serotypes; NVT, non-vaccine serotypes; τ^2, the absolute variance in the true effect sizes across studies; I2, the estimated between-study heterogeneity.

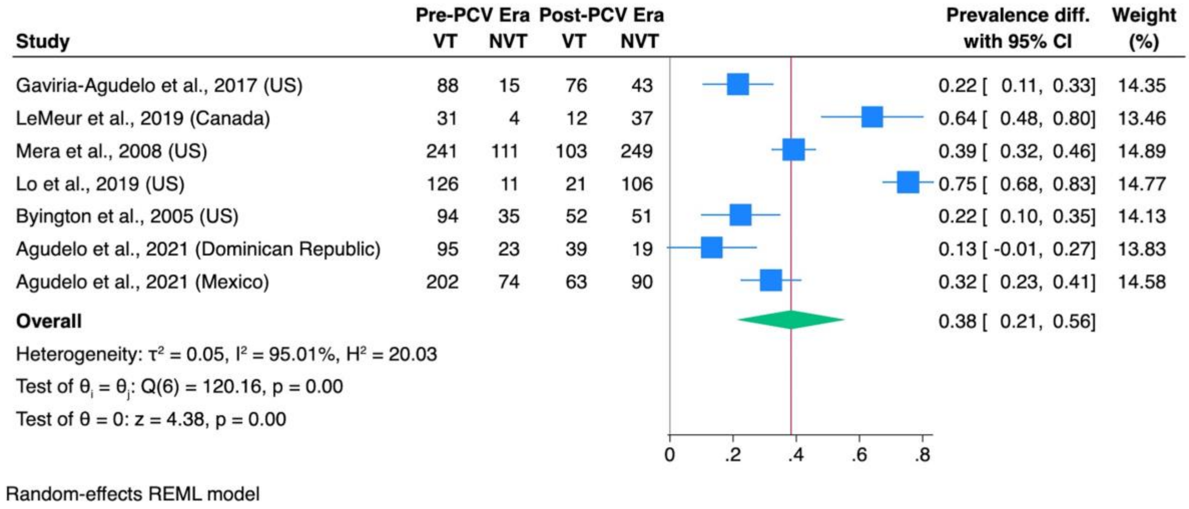

Fig. S4: Forest plot and meta-analysis for serotype prevalence pre and post PCV era in North America. For each study, the estimated prevalence difference with the corresponding 95% confidence interval and the result of random-effects meta-analysis, are shown. VT, vaccine serotypes; NVT, non-vaccine serotypes; τ^2, the absolute variance in the true effect sizes across studies; I2, the estimated between-study heterogeneity.

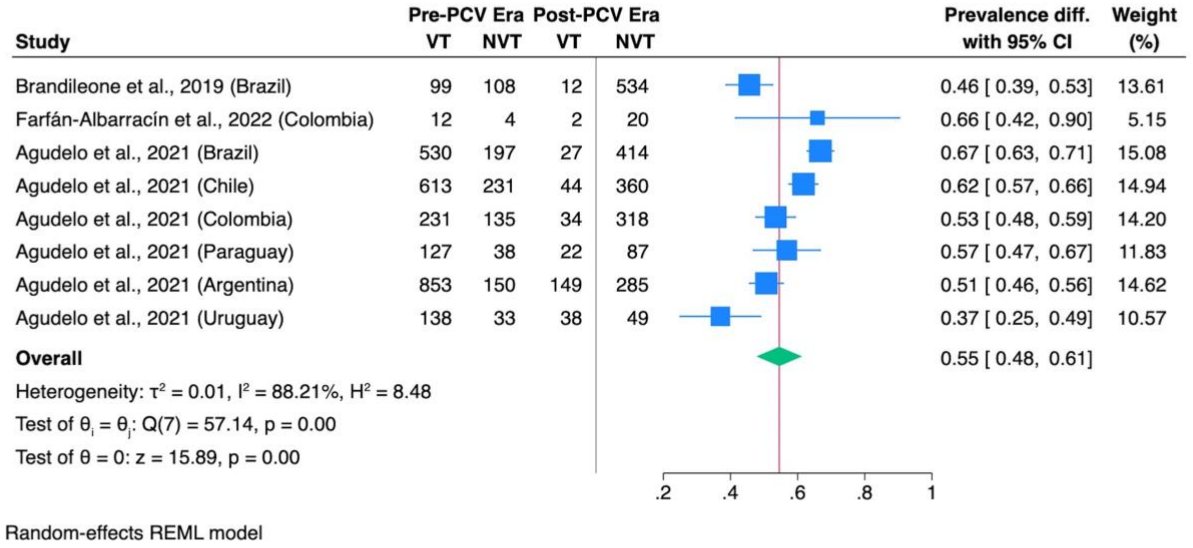

Fig. S5: Forest plot and meta-analysis for serotype prevalence pre and post PCV era in South America. For each study, the estimated prevalence difference with the corresponding 95% confidence interval and the result of random-effects meta-analysis, are shown. VT, vaccine serotypes; NVT, non-vaccine serotypes; τ^2, the absolute variance in the true effect sizes across studies; I2, the estimated between-study heterogeneity.

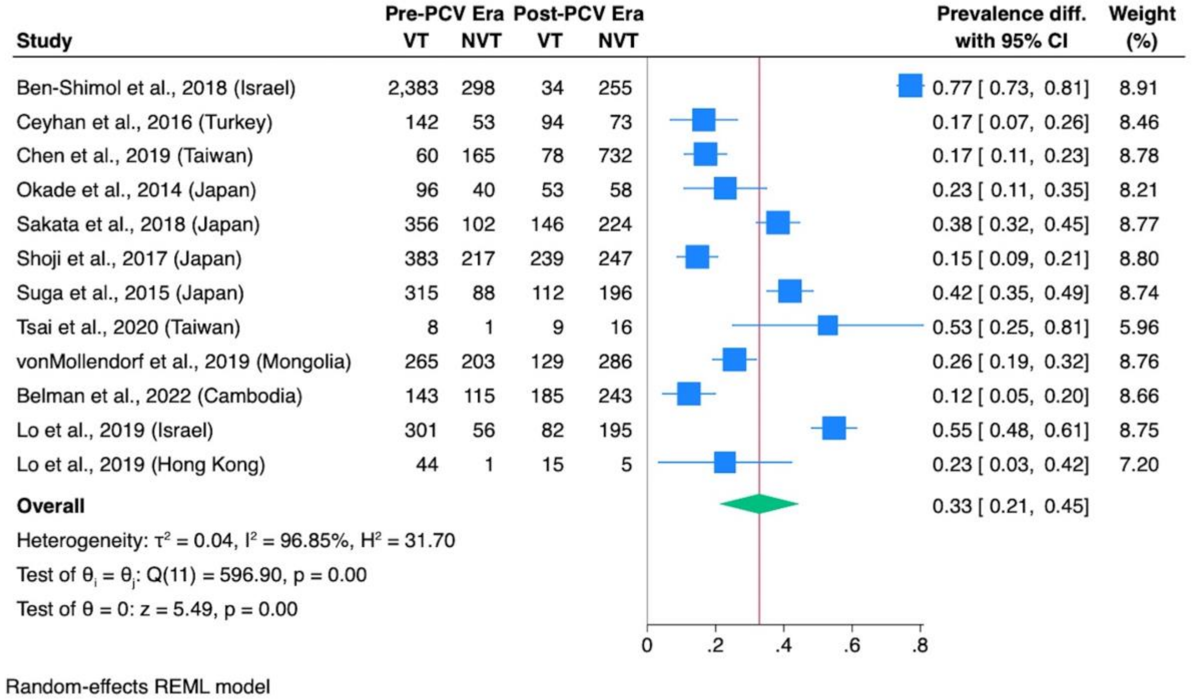

Fig. S6: Forest plot and meta-analysis for serotype prevalence pre and post PCV era in Asia. For each study, the estimated prevalence difference with the corresponding 95% confidence interval and the result of random-effects meta-analysis, are shown. VT, vaccine serotypes; NVT, non-vaccine serotypes; τ^2, the absolute variance in the true effect sizes across studies; I2, the estimated between-study heterogeneity.

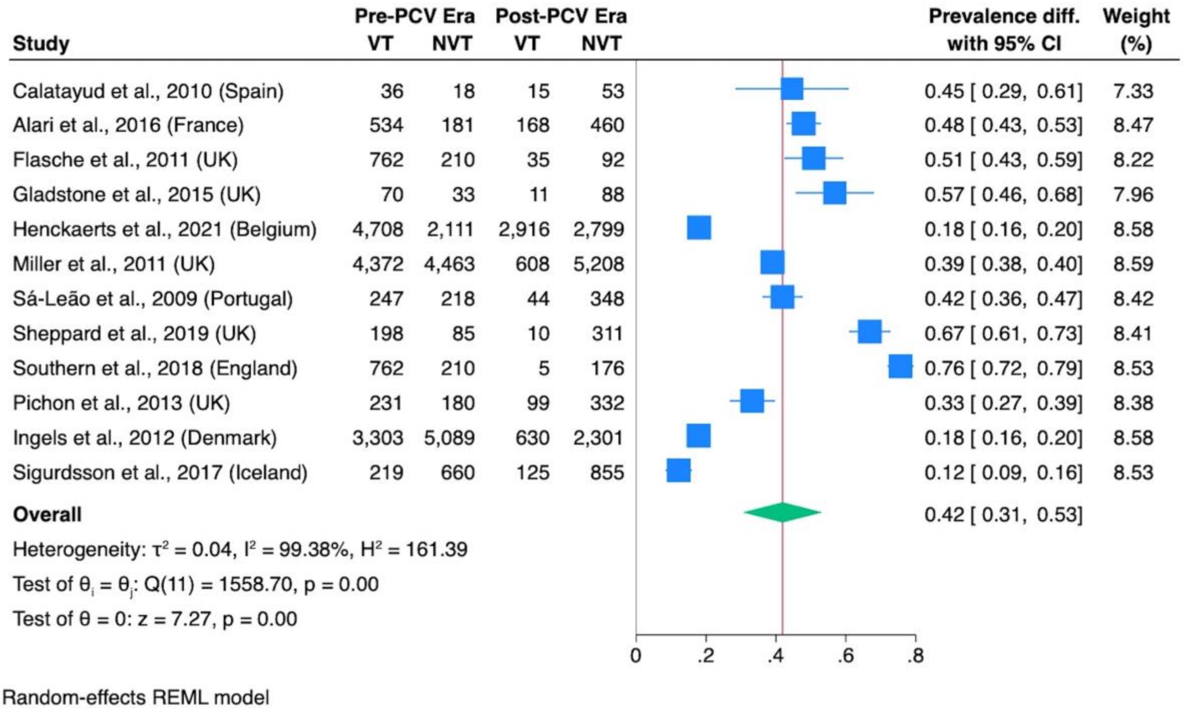

Fig. S7: Forest plot and meta-analysis for serotype prevalence pre and post PCV era in Europe. For each study, the estimated prevalence difference with the corresponding 95% confidence interval and the result of random-effects meta-analysis, are shown. VT, vaccine serotypes; NVT, non-vaccine serotypes; τ^2, the absolute variance in the true effect sizes across studies; I2, the estimated between-study heterogeneity.

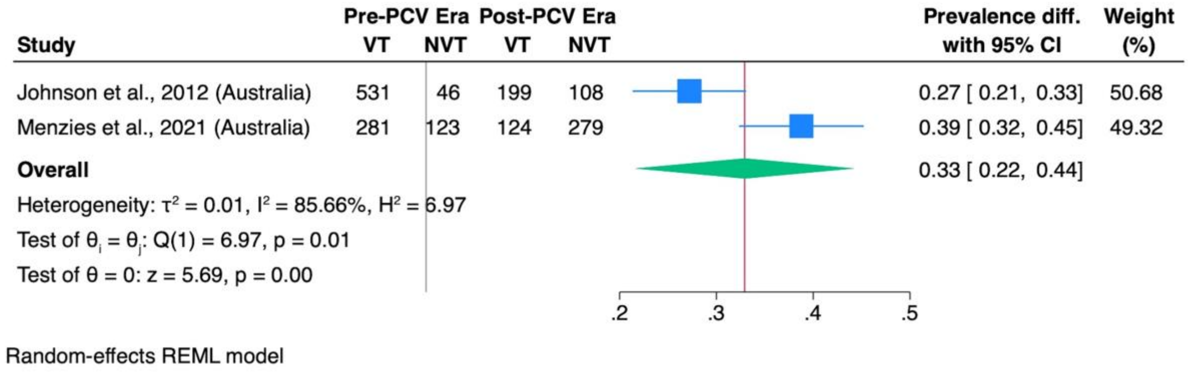

Fig. S8: Forest plot and meta-analysis for serotype prevalence pre and post PCV era in Oceania. For each study, the estimated prevalence difference with the corresponding 95% confidence interval and the result of random-effects meta-analysis, are shown. VT, vaccine serotypes; NVT, non-vaccine serotypes; τ^2, the absolute variance in the true effect sizes across studies; I2, the estimated between-study heterogeneity.
